# Chronic Memantine Treatment Ameliorates Behavioral Deficits, Neuron Loss, and Impaired Neurogenesis in a Model of Alzheimer’s Disease

**DOI:** 10.1007/s12035-020-02120-z

**Published:** 2020-09-10

**Authors:** Martina Stazi, Oliver Wirths

**Affiliations:** Department of Psychiatry and Psychotherapy, Molecular Psychiatry, University Medical Center (UMG), Georg-August University, Von-Siebold-Str. 5, 37075 Göttingen, Germany

**Keywords:** Alzheimer, Memantine, Neuron loss, Transgenic mice, Amyloid, Behavior

## Abstract

Memantine, a non-competitive NMDA receptor antagonist possessing neuroprotective properties, belongs to the small group of drugs which have been approved for the treatment of Alzheimer’s disease (AD). While several preclinical studies employing different transgenic AD mouse models have described beneficial effects with regard to rescued behavioral deficits or reduced amyloid plaque pathology, it is largely unknown whether memantine might have beneficial effects on neurodegeneration. In the current study, we assessed whether memantine treatment has an impact on hippocampal neuron loss and associated behavioral deficits in the Tg4-42 mouse model of AD. We demonstrate that a chronic oral memantine treatment for 4 months diminishes hippocampal CA1 neuron loss and rescues learning and memory performance in different behavioral paradigms, such as Morris water maze or a novel object recognition task. Cognitive benefits of chronic memantine treatment were accompanied by an amelioration of impaired adult hippocampal neurogenesis. Taken together, our results demonstrate that memantine successfully counteracts pathological alterations in a preclinical mouse model of AD.

**Electronic supplementary material:**

The online version of this article (10.1007/s12035-020-02120-z) contains supplementary material, which is available to authorized users.

## Introduction

Alzheimer’s disease (AD) is an irreversible, chronic, and progressive neurodegenerative disorder representing the most common cause of dementia [1]. AD is associated with a gradual decline in memory and cognition, linked to neuronal death in the cerebral cortex and limbic system of the brain [2]. Until now, no disease-modifying treatments are available, and current pharmacotherapeutic strategies are based on symptom relief, preservation of mental abilities, and potential delay in the progression of neurodegeneration. A limited number of drugs are approved by the regulatory authorities which can be classified into two main categories: (1) acetylcholinesterase inhibitors, which prevent the break-down of the neurotransmitter acetylcholine into choline and acetate and are approved to treat mild to moderate dementia, and (2) glutamatergic antagonists, which are intended to protect nervous tissue against glutamate-mediated excitotoxicity [3]. Glutamate is the major excitatory neurotransmitter in the brain, and glutamatergic overstimulation might result in neuronal damage and ultimate cognitive decline. Memantine, a non-competitive N-methyl-D-aspartate receptor (NMDAR) antagonist of moderate affinity, is the only member of this class of drugs approved to treat moderate to severe AD [4]. The idea of using memantine in AD is based on the observation that blocking NMDAR and concomitant reduction of their over-excitation can preserve neurons and their functions [5, 6]. There is plenty of evidence from in vitro [7–9] and in vivo studies [10–12], showing that memantine protects neurons from the toxic effects of glutamate by restoring glutamatergic system homeostasis [6]. In addition, a variety of studies using classical mutant amyloid precursor protein (APP) overexpressing mouse models of AD have described an amelioration of behavioral deficits and reduced β-amyloid (Aβ) pathology [13–17]. However, there is currently no published data from post-mortem studies available, addressing a potential beneficial effect of memantine with regard to a slowdown of neuronal degeneration in AD patients. The aim of this study was an evaluation of the potential effects of chronic memantine treatment in the Tg4-42 mouse model of AD, which is characterized by age-dependent neuron loss in the CA1 region of the hippocampus together with robust behavioral deficits [18, 19]. In contrast to most of the previously used AD mouse models in which effects of memantine treatment have been analyzed, these mice do not overexpress mutant forms of APP and might be more relevant for the sporadic form of the human disease [20]. We previously demonstrated that this mouse model presents with reduced neurogenesis in the dentate gyrus (DG) of the hippocampus in comparison with age-matched WT animals [21]. In the current study, homozygous Tg4-42 and WT control mice were orally treated with the NMDA receptor antagonist memantine. The treatment started at the age of 2 months, prior to the occurrence of neuron loss and behavioral deficits, lasting for a period of 4 months, exceeding the treatment period of most other studies. At 6 months of age, a battery of anxiety, motor, and memory tests as well as an evaluation of neuron loss and neurogenesis were carried out. Here, we demonstrate that long-term memantine treatment decreases CA1 neuron loss, partially ameliorates motor deficits, and rescues memory impairment in different behavioral paradigms.

## Material and Methods

### Mice

Generation of the Tg4-42 mouse model has been published previously [18]. In brief, the Tg4-42 mouse model utilizes the murine Thy1 promotor to overexpress a genetic construct comprising the human Aβ_4-42_ sequence fused to the murine thyrotropin-releasing hormone (TRH) signal peptide, allowing Aβ secretion. Tg4-42 mice were generated and maintained on a C57Bl/6J genetic background. In this study, homozygous Tg4-42 (Tg4-42^hom^) and C57Bl/6J mice (WT) (Jackson Laboratories, Bar Harbor, ME, USA) were used. All animals were handled according to the German guidelines for animal care, and all experiments have been approved by the local animal care and use committee (Landesamt für Verbraucherschutz und Lebensmittelsicherheit (LAVES), Lower Saxony). In this study, an equal number of female and male mice were used. Access to food and water was provided ad libitum.

### Drug Treatment

**Fig. 1:**
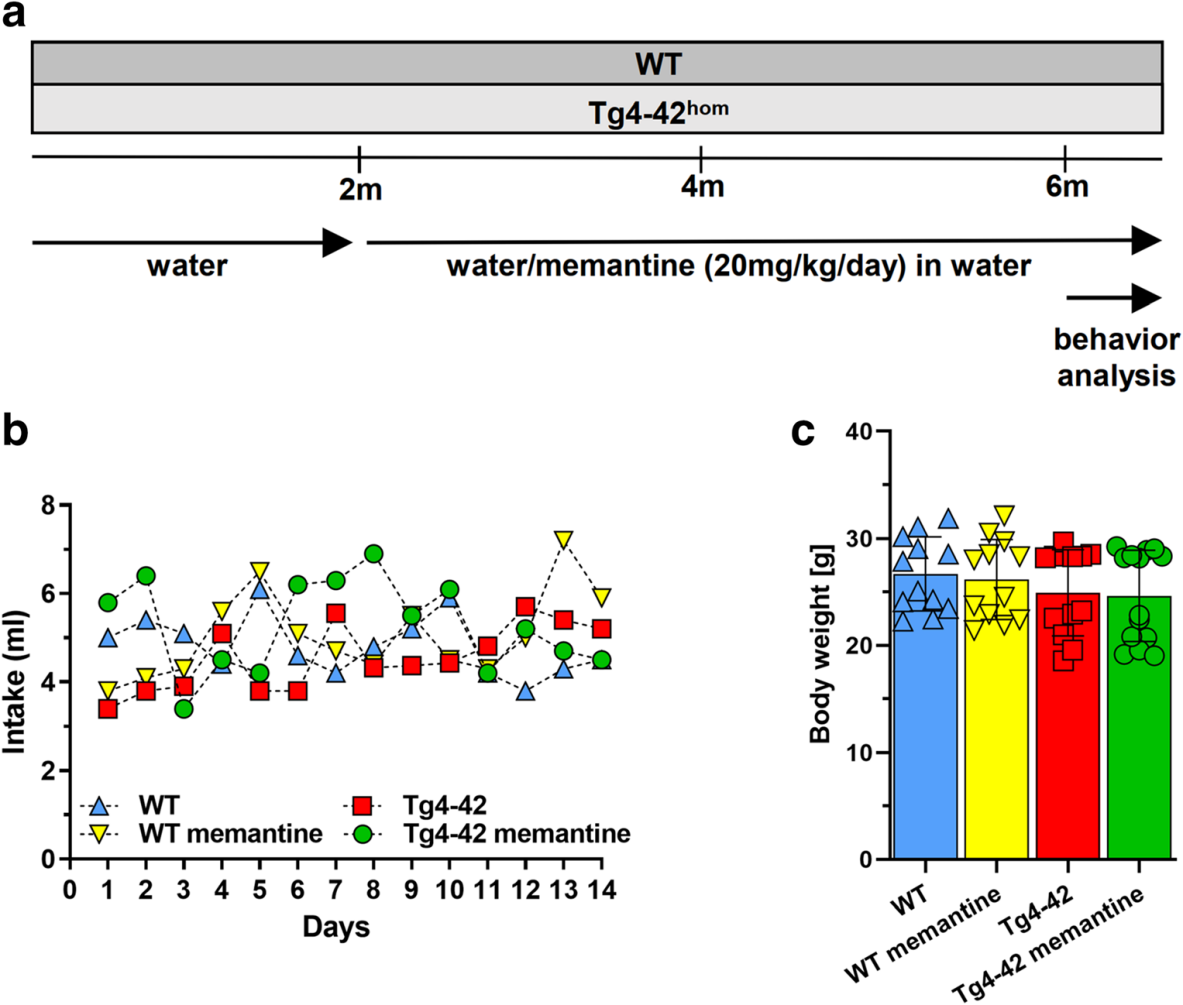
Two-month-old wild type (WT) and homozygous Tg4-42 (Tg4-42^hom^) mice were treated for 4 months with memantine (20 mg/kg/day) in drinking water. At 6 months of age, mice were subjected to a battery of behavioral tests for a duration of 2 weeks with ongoing treatment (**a**). Daily water consumption and body weight assessment during the behavioral test analysis. The daily water intake was similar between the groups (**b**), and memantine treatment does not have an influence on the body weight of mice (**c**). Two-way repeated measures analysis of variance (ANOVA) or one-way ANOVA followed by Bonferroni multiple comparisons tests. Data are presented as mean ± SD

Long-term oral memantine treatment was initiated at 2 months of age (Fig. 1a). Memantine hydrochloride (Heumann, 10 mg/ml) was administered orally via drinking water, at a daily dose of 20 mg/kg for 4 months, and was maintained during the behavioral test phase. Oral memantine administration of 20–30 mg/kg/day has been successfully demonstrated to result in clinically relevant constant plasma levels [13] and has been shown to have neuroprotective effects together with memory improvement in mouse models of AD [13, 22, 23]. Water consumption was measured daily throughout the behavioral analysis to confirm average daily memantine intake in all treatment groups, including the control groups (WT and Tg4-42^hom^) receiving tap drinking water.

### Behavioral Testing

To detect potential beneficial effects of prolonged memantine treatment with regard to learning and behavior, male and female WT and Tg4-42 mice in an equal gender distribution were tested at 6 months of age at the end of the treatment period in a battery of anxiety, motor, and memory tests (*n* = 11–15 per group). Animals were kept on a 12-h/12-h inverted dark/light cycle (light phase between 8 p.m. and 8 a.m.), and mice were sacrificed immediately after the last day of testing. All behavior experiments were performed during the dark phase.

### Accelerating Rotarod

Motor performance, motor learning, and balance skills were examined using the accelerating rotarod test [24] (RotaRod, TSE Systems GmbH, Bad Homburg, Germany). The test was carried out under red light conditions for two consecutive days with 4 trials per day and at least 15-min inter-trial intervals. Each mouse was individually placed on the rod, which accelerates from 4 to 40 revolutions per minute (rpm) over a maximal trial time of 300 s. Trials were completed when animals fell off or the maximum time was reached, and latency to fall (s) was recorded as an indicator of motor performance. The apparatus was cleaned between trials with 70% ethanol to avoid odor cues.

### Balance Beam

The balance beam was used to evaluate fine motor coordination and balance of the mice as described previously [25]. A beam (50 cm long and ø 1 cm) was clamped between two escape platforms (9 × 15 cm) 44 cm above a padded surface. The mouse was placed in the middle of the beam, facing one of the two platforms, and the latency to fall from the beam was recorded as the average of three 60-s trials. Mice were allowed to rest for at least 10 min between each trial. If the mouse escaped to one of the platforms or remained on the beam throughout the trial, the maximum time (60 s) or otherwise the latency to fall was recorded. Between the trials, the apparatus was cleaned with 70% ethanol to diminish odor cues.

### Elevated Plus Maze

The elevated plus maze (EPM) was used to assess exploratory behavior and anxiety levels [25]. In brief, the EPM consisted of four arms of 15-cm length and 5-cm width in a “+” configuration, raised 75 cm above a padded surface. Two oppositely positioned arms contained lateral walls (closed arms), whereas the other pair of arms were opened (open arms). Experiments were carried out under red light conditions, and mice were placed in the central area of the apparatus facing one of the open arms. Mice were allowed to explore the maze freely for 5 min. Distance traveled, average speed, arm entries, and percentage of time spent in each arm were recorded and calculated using the ANY-Maze tracking software (Stoelting Europe, Ireland, Dublin). Anxiety-like behavior was calculated using the time spent in the open arms, with longer times spent in the open arms corresponding to reduced anxiety levels [26]. The EPM was cleaned after each mouse using 70% ethanol to eliminate odor cues.

### Open Field and Novel Object Recognition

The open field (OF) test was used to assess locomotor activity, exploratory behavior, and anxiety levels. During the OF test, mice were placed in the middle of a squared arena (50 × 50 cm) where they could freely explore the environment for 5 min. During single 5-min trials, the total time spent in the central part of the arena, the total distance traveled, and the average speed were recorded using a video tracking software (ANY-maze, Stoelting Europe). Twenty-four hours after the OF, the novel object recognition test (NOR) was performed in the same box, now including two identical objects (exploration phase). The NOR is a widely used test to assess recognition memory and novelty preference in rodents [27]. Mice were allowed to freely explore the objects for 5 min and were returned to their homecage. Twenty-four hours later, one of the objects was exchanged with a novel object consistent in height and volume but different in shape and appearance (testing phase). Object exploration was scored whenever the mouse sniffed or touched the objects when looking at them, while climbing onto the object was not rated as exploration [28]. Data collection and video analysis were performed blind to the experimental condition and were carried out by two individual experimenters to assess reproducibility. The percentage of exploration time for the novel object was calculated as follows: (time novel object × 100)/(time novel object + time familiar object).

In addition, observation scores on day 2 were converted into discrimination indices (DI) to determine novel versus familiar object exploration rates: (time novel object − time familiar object)/(time novel object + time familiar object).

In between trials, the arena as well as the objects was cleaned with 70% ethanol to diminish odor cues.

### Morris Water Maze

The Morris water maze [29] (MWM) test was used to assess spatial reference memory as previously described [18]. Briefly, mice are trained to learn to localize a submerged platform (ø 10 cm) in a circular pool (ø 110 cm) filled with non-toxic white paint. The pool was divided into four virtual quadrants relating to the platform position, which were designated left (L), right (R), opposite (O), and target (T) quadrants. Initially, a “cued training,” was carried out for three consecutive days, in which the submerged platform was marked with a triangular flag. In the “acquisition training,” with a duration of 5 days, the triangular flag was removed and proximal cues were added around the pool. During the final “probe trial,” the platform was removed; however, proximal and distal cues remained. Since the platform location was kept constant during the acquisition training, mice that have successfully acquired spatial reference memory should demonstrate a target quadrant preference. Between the trials, mice were dried and kept under infrared light to prevent hypothermia. All trials were recorded using a video tracking software (ANY-maze, Stoelting Europe), and parameters such as escape latency, swimming speed, swimming path, and quadrant preference were extracted.

### Tissue Collection and Preservation

Brain tissues were collected and preserved in different ways depending on the following analysis. Mice were deeply anesthetized and transcardially perfused using ice-cold 0.01 M phosphate-buffered saline (PBS), and brains were carefully dissected. The right hemisphere was post-fixed in 4% formalin solution at 4 °C for at least 72 h protected from light before embedding in paraffin. The left hemisphere was post-fixed in 4% paraformaldehyde (PFA) in 0.01 M PBS for at least 24 h before being transferred to a 30% sucrose solution (in 0.01 PBS) for cryo-protection. Next, brain tissue was deep-frozen on a dry ice plate and stored at − 80 °C until further use.

### Quantification of CA1 Neuron Numbers

Neuronal loss in the CA1 region of the hippocampus was assessed on 4-μm sagittal paraffin brain sections (Bregma 1.08–1.32) cut on a rotation microtome (Microm, HM335E, Thermo Fisher Scientific, Germany) and stained with hematoxylin [30]. Neuronal nuclei were determined by their size and peculiar appearance clearly differing from glial cells. Images of the CA1 area of the hippocampus were acquired at × 400 magnification using an Olympus BX-51 microscope equipped with a Moticam pro 282 camera (Motic, Wetzlar, Germany). The number of CA1 neurons per section (*n* = 3 per animal) was counted using the manual cell-counting tool implemented in ImageJ (version 1.52u, NIH). The CA1 layer was separated into proximal (extending to CA2) and distal (towards subiculum) parts, and relative results setting the WT group as a reference were calculated. The experimenter was blinded with regard to genotype and treatment throughout all the analysis.

### Analysis of Adult Neurogenesis

Frozen cryo-protected brain hemispheres were cut into series of 30-μm-thick coronal sections using a cryostat (CM1850 UV, Leica, Germany). A series of every 10th coronal frozen section was processed in a free-floating staining protocol to quantify the number of new-born neurons. First, one brain section series was rehydrated for 10 min with ice-cold 0.01 M PBS, and endogenous peroxidase activity was blocked by immersion in 30% H_2_O_2_ in 0.01 M PBS for 30 min. Sections were washed in PBS containing 0.01% Triton X-100 for membrane permeabilization. Unspecific blocking was done for 1 h by incubation in 0.01 M PBS including 10% fetal calf serum (FCS) and 4% milk powder at room temperature (RT). Primary goat antibody against doublecortin (DCX, 1:500, Santa Cruz Biotechnology (sc-8066)) was diluted in 0.01 M PBS containing 10% FCS and incubated over night at RT. On the next day, sections were washed thoroughly with PBS including Triton X-100 and incubated with a secondary anti-goat biotinylated antibody (DAKO, Glostrup, Denmark). Staining was visualized using the ABC method using a Vectastain kit (Vector Laboratories, Burlingame, USA) and diaminobenzidine as chromogen. The total number of new-born neurons was counted in the DG using a stereology workstation and the meander scan option of Stereo Investigator 7 (MicroBrightField, Williston, USA) to quantify all DCX-positive cells in a given section. The resulting neuron number was multiplied by 10 to obtain the total number of new-born neurons per hemisphere [31]. The experimenter was blinded with regard to genotype and treatment throughout the entire analysis.

To avoid possible bias due to gender-dependent differences in brain size, for the quantification of CA1 neuron numbers and adult neurogenesis, only female mice were used (6-month-old WT and Tg4-42^hom^, treated and untreated with memantine, *n* = 6 per group).

### Statistical Analysis

Differences between groups were tested with either one-way or two-way analysis of variance (ANOVA) followed by Bonferroni multiple comparisons tests or Mann–Whitney tests as indicated. All data is presented as mean ± standard error of the mean (SEM) or ± standard deviation of the mean (SD). Significance levels are determined as follows: *p < 0.05, **p < 0.01, ***p < 0.001. All statistics were calculated using GraphPad Prism version 8.4 for Windows (GraphPad Software, San Diego, CA, USA).

## Results

There were no differences in water consumption between the different groups during the behavioral test period (Fig. 1b). Body weight was recorded throughout this period, and no significant differences were detected among all groups irrespective of the treatment (Fig. 1c).

### Chronic Memantine Treatment Does Not Restore Decreased Anxiety Levels in Tg4-42^hom^ Mice

**Fig. 2:**
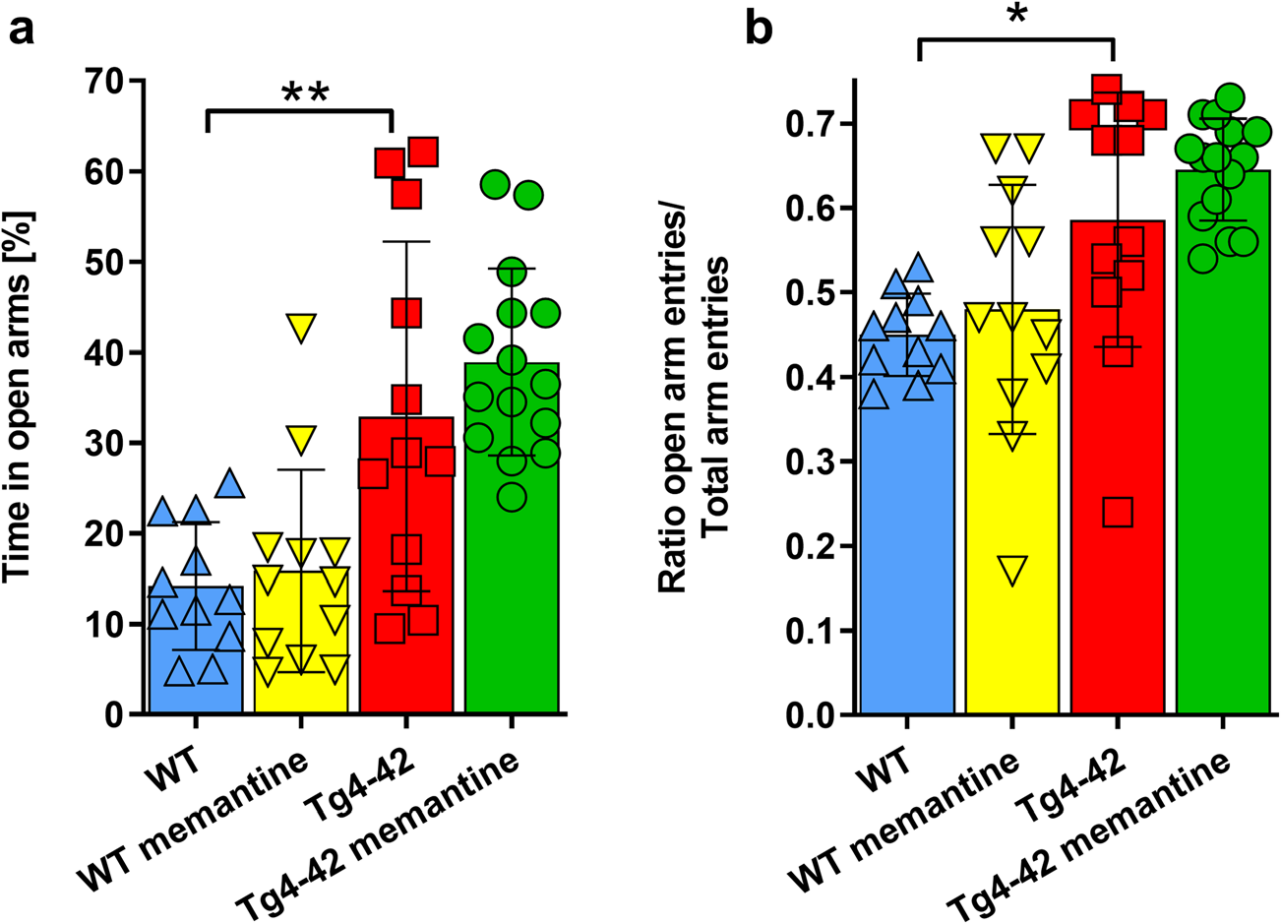
Chronic memantine treatment does not change the anxiety phenotype of Tg4-42^hom^ mice. **a**, **b** The elevated plus maze test revealed that untreated Tg4-42^hom^ mice show reduced anxiety levels, illustrated by significantly increased time spent in open arms compared with WT animals (**a**). All data were given as mean ± SD. *p < 0.05, **p < 0.01

While Tg4-42^hom^ mice performed indistinguishable from WT mice in the open field task (Supplemental Fig. S1), they showed significantly reduced anxiety levels compared with age-matched WT animals in the EPM paradigm (*p* < 0.01) (Fig. 2a). Chronic memantine treatment could not revert this altered anxiety phenotype, as shown by the percentage of time spent in the open arms (Fig. 2a). Calculating the ratio of open arm entries to total arm entries confirmed this result and revealed significantly higher ratios in Tg4-42^hom^ and Tg4-42^hom^ memantine-treated mice compared with WT control animals (*p* < 0.05 and *p* < 0.001 respectively) (Fig. 2b).

### Memantine Partially Ameliorates Motor Function in Tg4-42^hom^ Mice

**Fig. 3:**
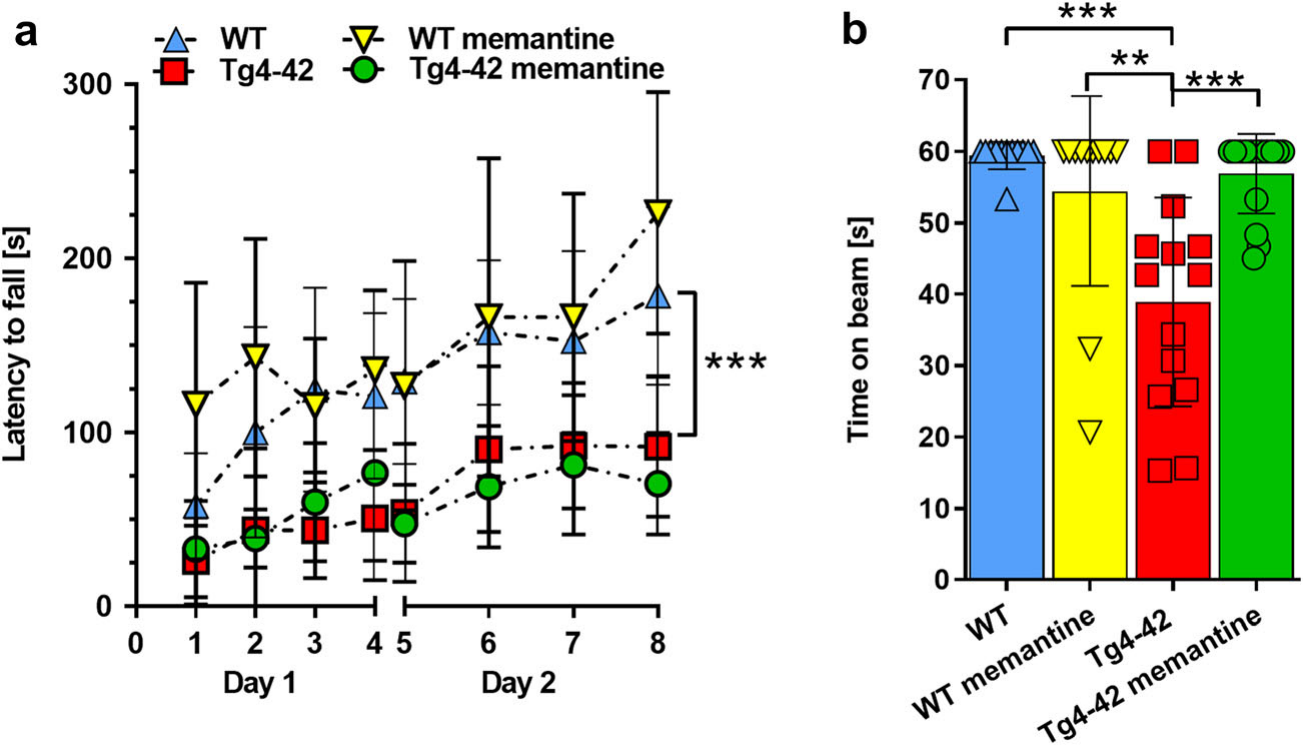
Memantine treatment has a partial effect on the motor performance of the Tg4-42^hom^ mice. **a** While Tg4-42^hom^ mice performed significantly worse compared with WT mice in the accelerating rotarod task, no improvement was detected upon chronic memantine treatment in Tg4-42^hom^ mice. **b** Memantine treatment showed a beneficial effect on motor performance in the balance beam task, with the memantine-treated Tg4-42^hom^ mice performing at WT levels. All data were given as mean ± SD. ***p < 0.001

After 4 months of chronic memantine treatment, motor performance was evaluated using the rotarod and the balance beam tasks (Fig. 3). In the rotarod test, balance and motor learning skills are analyzed. While WT and memantine-treated WT mice showed an improvement in their ability to stay on the rod over the trial period, aged-matched Tg4-42^hom^ showed a significant impairment in motor performance compared with WT animals, as shown by overall lower latencies to fall (Fig. 3a; *p* < 0.001 vs WT). This genotype-dependent motor deficit could not be rescued by memantine treatment (Fig. 3a). Memantine treatment did not influence genotype-dependent motor performance in the rotarod task or speed in the open field task (Suppl. Fig. S1B), suggesting that memantine does not impact general physical activity. Moreover, Tg4-42^hom^ performed significantly worse than the aged-matched WT group in the balance beam task (*p* < 0.001). However, this phenotype could be rescued after 4 months of memantine treatment as shown by the significantly increased latency to fall in drug-treated Tg4-42^hom^ mice (*p* < 0.001) (Fig. 3b).

### Memantine Restores Object Recognition and Spatial Memory in Tg4-42^hom^ Mice

**Fig. 4:**
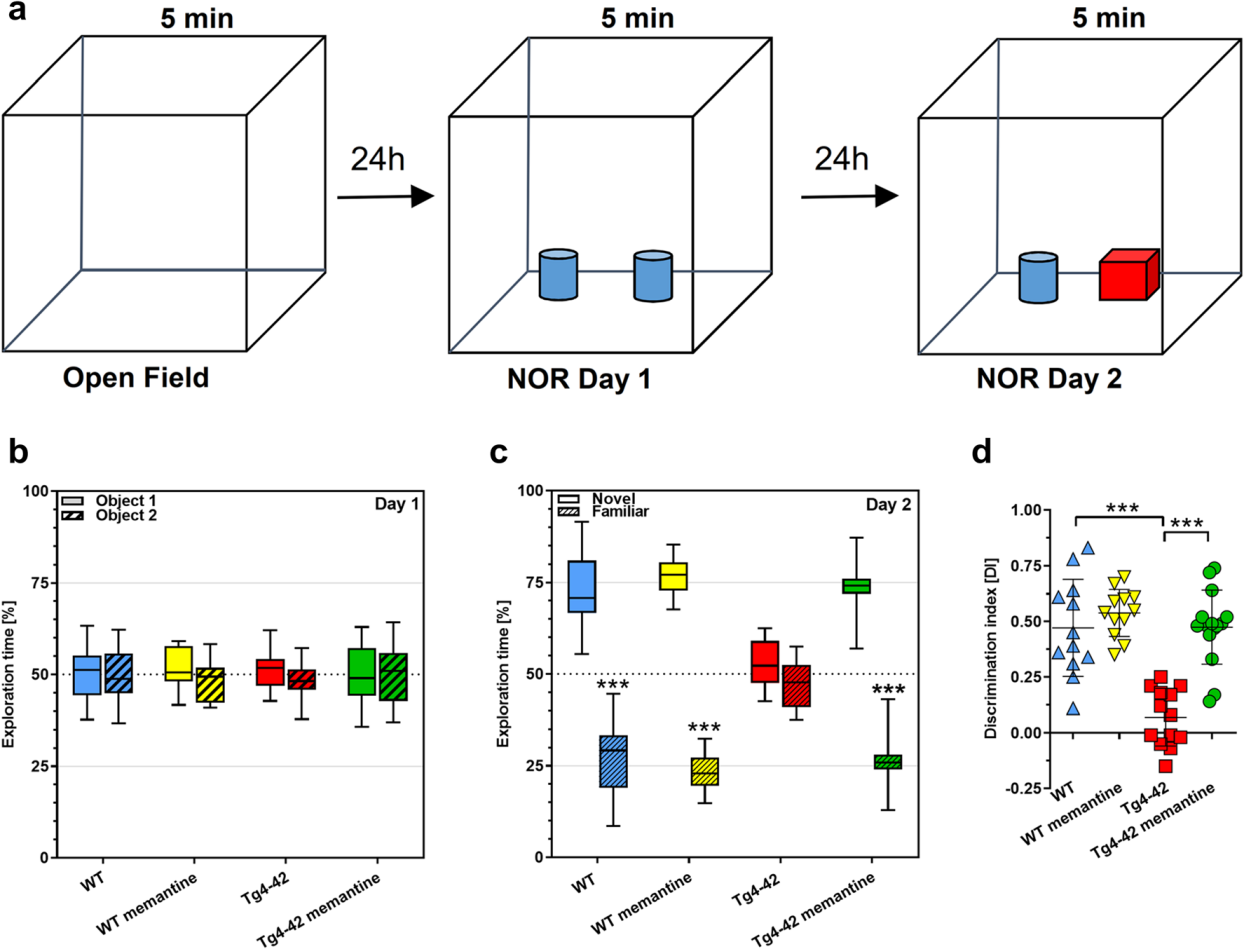
Chronic memantine treatment rescues impaired object recognition memory in Tg4-42^hom^ mice. **a** Simplified scheme of the NOR test. **b** During the exploration phase, all groups spent ~ 50% of time exploring each of the two similar objects. **c** On the testing day, Tg4-42^hom^ untreated mice showed no object preference, while all other groups revealed a significant preference for the novel object. **d** Novel object recognition performance, as shown by DI, indicates an inability of Tg4-42^hom^ mice to discriminate object novelty, with significantly lower DI compared to all other groups. Two-way ANOVA followed by Bonferroni’s post hoc test. All data were given as mean ± SD. ***p < 0.001

Recognition memory was evaluated using the novel object recognition task (Fig. 4a). During the exploration phase on day one, all groups spent an equal amount of time exploring each of the similar objects (Fig. 4b). After a 24-h delay on the testing day, untreated Tg4-42^hom^ mice spent an equal amount of time exploring the familiar and novel objects, while the untreated WT animals, as well as drug-treated WT and Tg4-42^hom^ mice, spent significantly more time exploring the novel object (*p* < 0.001), indicating that they were able to discriminate between the novel and familiar objects (Fig. 4c). Calculation of the DI confirmed this observation, with Tg4-42^hom^ untreated mice showing a significantly lower DI compared to all other groups (*p* < 0.001) (Fig. 4d).

**Fig. 5:**
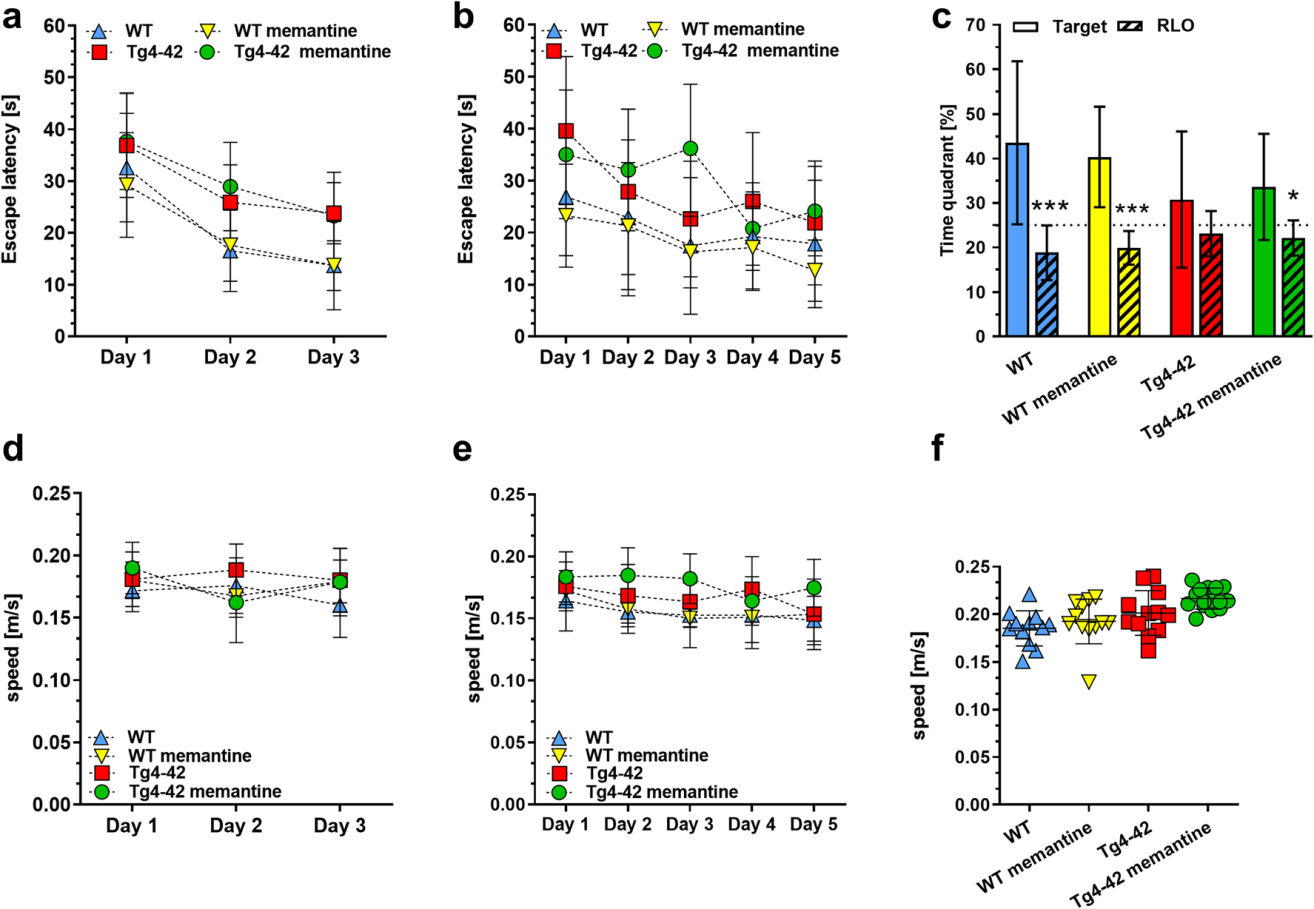
Memantine treatment rescues impaired spatial memory performance in Tg4-42^hom^ mice. **a**, **b** All groups showed progressively reduced escape latencies during the cued and acquisition training. **c** While Tg4-42^hom^ showed no preference for any of the quadrants during the probe trial, WT controls, memantine-treated WT, and memantine-treated Tg4-42^hom^ mice spent significantly more time in the target quadrant (T) compared with all the other quadrants (RLO), disclosing intact spatial reference memory. **d**–**f** No differences in swimming speed were observed within all the groups in cued training, acquisition training, and probe trial. **a**–**e** Two-way repeated measures ANOVA followed by Bonferroni multiple comparisons. **f** One-way ANOVA followed by Bonferroni’s multiple comparisons test. *p < 0.05, ***p < 0.001. All data were given as mean ± SEM (T, target; L, left; R, right; O, opposite)

Spatial reference memory was evaluated in drug-treated and untreated mice using the MWM task. All groups showed progressively decreased escape latencies over 3 days of cue training (Fig. 5a). No differences in swimming speed were observed (Fig. 5d), indicating intact vision and swimming skills in all groups. During the following 5 days of acquisition training, all mice, regardless of treatment, displayed gradually decreased escape latencies, and again no difference in swimming speed was detected (Fig. 5b and e respectively). During the probe trial, carried out 24 h after the last acquisition trial, untreated Tg4-42^hom^ mice showed no preference for the target quadrant. In contrast, WT control mice, memantine-treated WT, and memantine-treated Tg4-42^hom^ mice spent significantly more time in the target quadrant compared with the average time spent in the other three pool quadrants (*p* < 0.0001 and *p* < 0.05 respectively) (Fig. 5c). This indicates a rescue of spatial reference memory in drug-treated Tg4-42^hom^ mice, as no differences in swimming speed were observed in the probe trial (Fig. 5f). Representative occupancy plots confirmed that untreated Tg4-42^hom^ mice showed a more random search strategy, while the other groups disclosed a more focused search of the platform position in the target quadrant (Supplementary Fig. S2).

### Memantine Ameliorates Hippocampal Neuronal Loss in the CA1 Area of Tg4-42^hom^ Mice

**Fig. 6:**
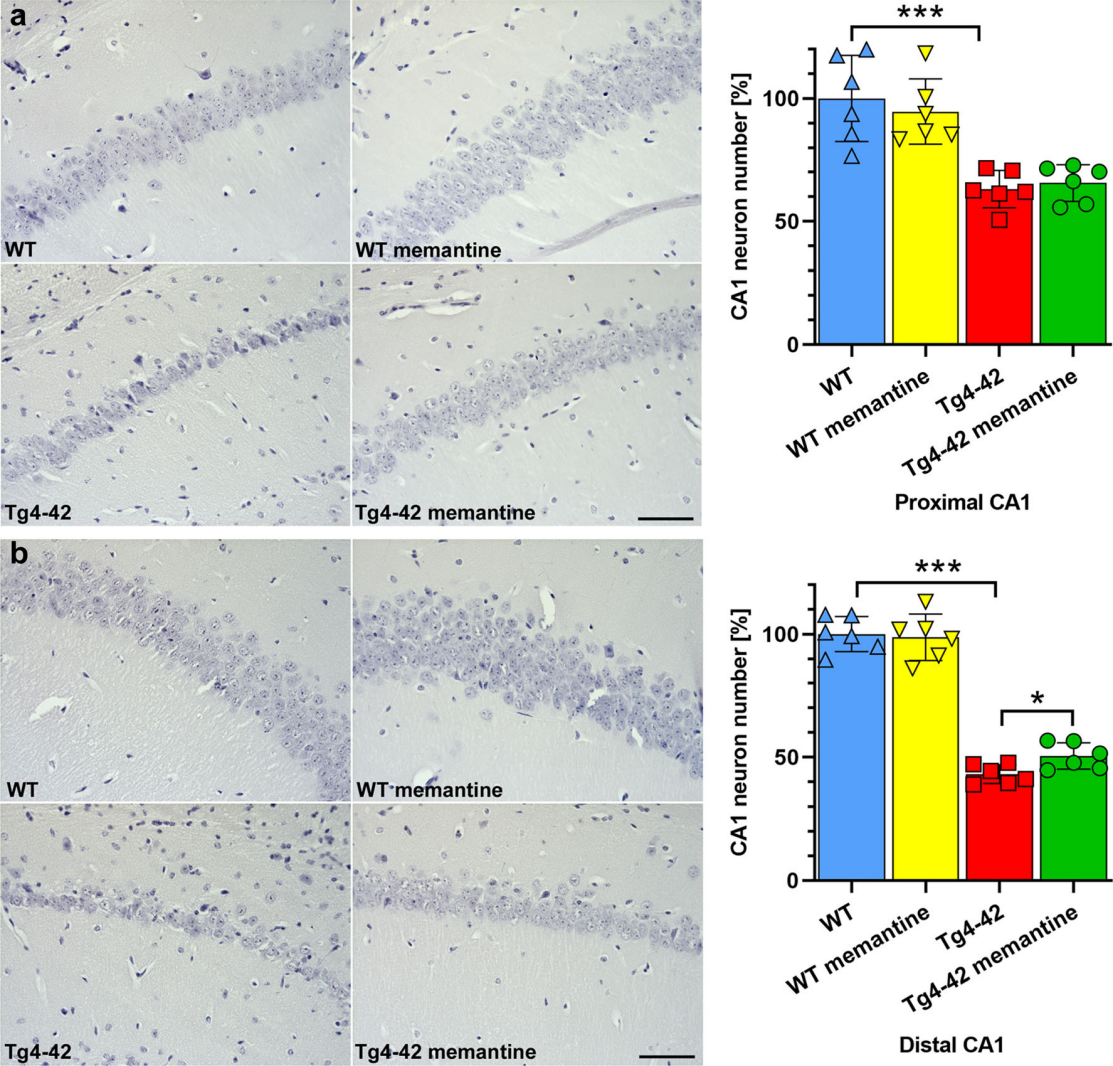
Memantine treatment decreases neuron loss in the CA1 region of the hippocampus of the Tg4-42^hom^ mice. **a** Analysis of hematoxylin-stained sections revealed significantly reduced proximal CA1 neuron numbers in Tg4-42^hom^ compared to WT mice. **b** This massive neuron loss was reduced in the distal CA1 upon drug treatment as Tg4-42^hom^ memantine mice showed significantly higher CA1 neuron numbers when compared to Tg4-42^hom^ untreated littermates. One-way ANOVA followed by the Mann–Whitney test. All data were given as mean ± standard deviation (SD). *p < 0.05, ***p < 0.001, scale bar: 50 μm

As 6-month-old Tg4-42^hom^ mice present a drastic loss of CA1 pyramidal neurons, we examined whether chronic memantine treatment might exert neuroprotective properties. Therefore, we quantified the number of hematoxylin-stained neuronal nuclei in the hippocampal CA1 region in 6-month-old untreated and drug-treated WT and Tg4-42^hom^ mice. An analysis discriminating between the proximal (extending to CA2) and the distal (towards subiculum) parts of the CA1 layer revealed no difference among treated and untreated WT mice. In contrast, a non-significant amelioration of ~ 4% in the proximal and a more pronounced ~ 17% amelioration of neuron loss in the distal CA1 part among Tg4-42^hom^ and Tg4-42^hom^ memantine-treated animals (*p* < 0.05; Fig. 6) could be detected. Compared with the untreated WT control group, Tg4-42^hom^ mice displayed a neuron loss of ~ 37% in the proximal CA1 and ~ 57% in the distal CA1 (Fig. 6), which is in good agreement with previous results showing an ~ 50% overall CA1 neuron loss compared with age-matched WT mice [19]. A correlation analysis between the discrimination index in the NOR and CA1 neuron numbers revealed a highly significant correlation for the distal (Pearson *r* = 0.7189, *p* = 0.0084) but no correlation for the proximal CA1 part (Pearson *r* = 0.2719, *p* = 0.3926) (Supplemental Fig. S3).

### Memantine Ameliorates Impaired DG Neurogenesis

**Fig. 7:**
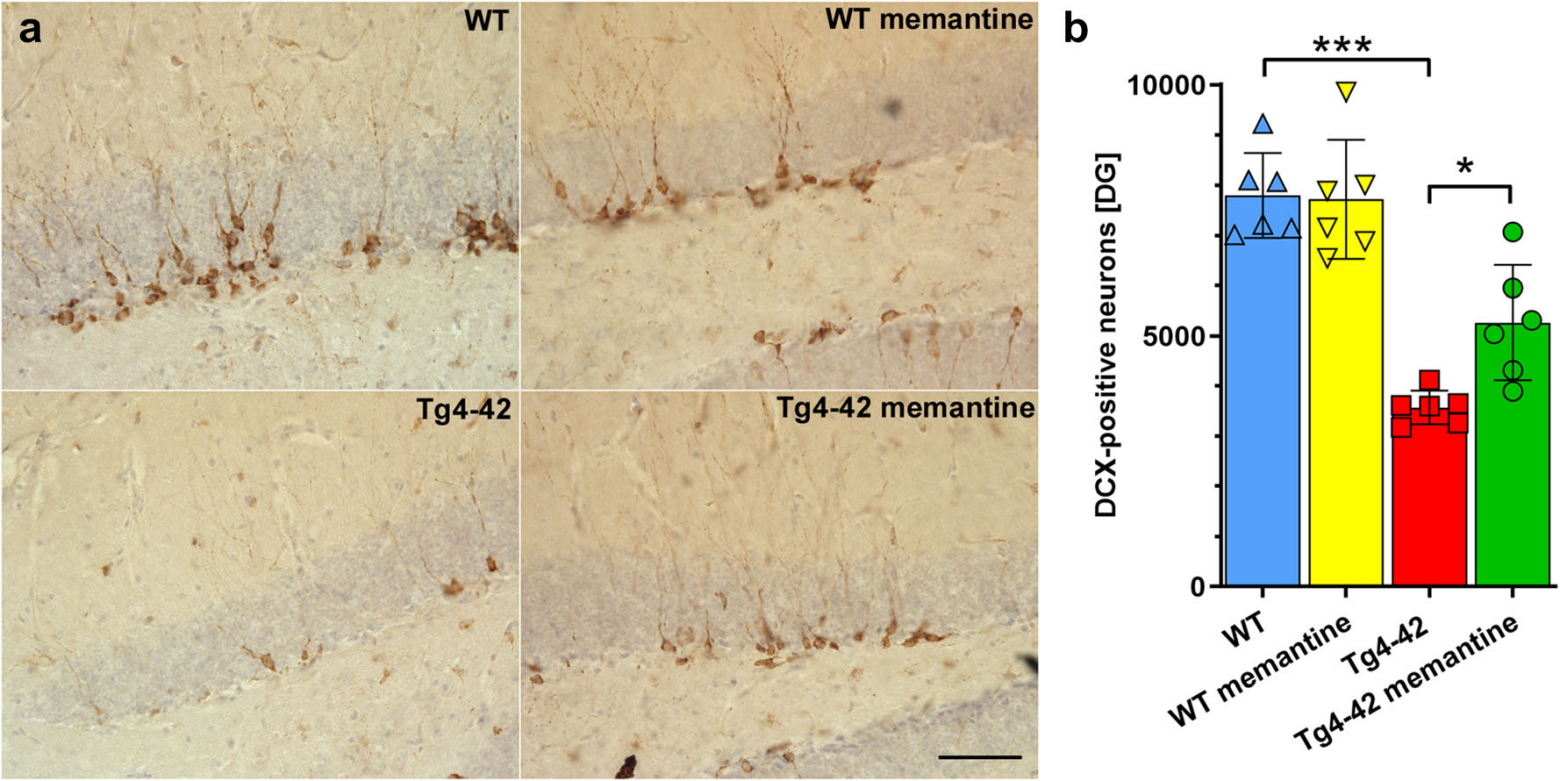
Chronic oral memantine treatment ameliorates impaired neurogenesis in the DG of Tg4-42^hom^ mice. Analysis of doublecortin (DCX)-stained sections revealed a significantly reduced number of DCX-positive cells in Tg4-42^hom^ mice compared with untreated WT mice (**a**, **b**). While chronic memantine treatment did not alter the number of new-born cells in WT mice, a significantly increased number was detected in memantine-treated Tg4-42 mice. One-way ANOVA followed by Bonferroni’s multiple comparisons test. *p < 0.05, ***p < 0.001, scale bar: 50 μm

Compared with WT mice, Tg4-42^hom^ mice at 6 months of age showed a strongly reduced number of DCX-positive neurons in the DG of the hippocampus (*p* < 0.0001; Fig. 7). While chronic memantine treatment did not alter neurogenesis in WT mice, ~ 33% more DCX-positive neurons were detected in memantine-treated Tg4-42^hom^ mice compared with non-treated Tg4-42^hom^ littermates (*p* < 0.05; Fig. 7). A correlation analysis revealed a significant correlation between the neurogenesis rate and the performance in the NOR (Pearson *r* = 0.6389, *p* = 0.0253) (Supplemental Fig. S3).

## Discussion

So far, only two classes of drugs have been approved to treat AD, which are acetylcholinesterase inhibitors (such as rivastigmine, donepezil, or galantamine) and the NMDA receptor antagonist memantine [32]. While some of the acetylcholinesterase inhibitors are approved for all disease stages (e.g., donepezil or rivastigmine), memantine is only approved for moderate to severe dementia due to AD. Dementia treatment with either cholinesterase inhibitors or memantine seems to result in statistically significant, though clinically marginal, improvement in measures of cognition and global assessment of dementia [33, 34]. In the current report, we investigated the effects of chronic memantine treatment in the Tg4-42 mouse model of AD. This mouse model exclusively expresses the N-terminal truncated Aβ_4-42_ peptide, which is one of the most abundant Aβ variants in human brain (reviewed in [35]). These mice present with behavioral deficits in spatial and recognition memory tasks, reduced adult neurogenesis, and an age-dependent loss of CA1 pyramidal neurons [18, 21, 36].

A variety of previous studies investigated the effects of memantine treatment on neuropathological alterations as well as learning and memory deficits in transgenic AD mouse models [13–17, 23, 37–39]; however, differences in dosage, treatment duration, and the mouse models applied complicate to draw universal conclusions. Most of these models make use of *APP* and or *PSEN1* overexpression and harbor familial AD-associated mutations to achieve deposition of Aβ peptides in the form of extracellular plaques [40]. While these mice usually exhibited AD-related pathological alterations, mutant *APP* overexpression might cause additional phenotypic changes that are unrelated to AD [41]. *APP* overexpression in general also leads to an enhanced production of other APP fragments (such as C-terminal fragments), making it difficult to ascribe pathological features to Aβ or other generated fragments [42]. Moreover, memory impairment has been demonstrated in APP transgenic mice even without Aβ deposition, suggesting that other non-AD-related confounding factors might play a role [41, 42].

Administration of memantine in doses of 5 to 20 mg/kg for 6 months resulted in a significant decrease in amyloid plaque load in hippocampus and cortex of Tg2576 mice, however, without showing beneficial effects in a hippocampus-dependent contextual fear-conditioning task [23]. Short-term application for 4 weeks reduced brain levels of soluble and insoluble Aβ peptides in this model, and in vitro data suggested that memantine acts via reducing Aβ production through the regulation of intracellular APP trafficking [43]. In the widely used 5XFAD model of AD, memantine treatment with a daily dose of 10 mg/kg for a duration of 1 month led to significantly reduced amyloid plaque pathology in 3-month-old animals [44], while others found no effect on Aβ_42_ levels in 6–7-month-old 5XFAD mice [39]. APP/PS1 mice treated with intraperitoneal memantine injections of 10 mg/kg for 4 months also revealed significantly reduced amyloid plaque burden in cortex and hippocampus at the age of 7 months, together with significant improvements in a novel object recognition task test of short-term memory [14]. This is in good agreement with the results from the NOR in the current study, although Tg4-42 mice do not develop overt extracellular amyloid pathology.

As mentioned before, Tg4-42 mice harbor significant CA1 pyramidal neuron loss, and both spatial memory in the MWM [45] and object recognition memory in the NOR [46] seem to be critically dependent on CA1 integrity. It has been shown that especially CA1 pyramidal neurons towards the subiculum (distal CA1) are important for object recognition [47, 48] and that novel object exposure primarily activates the distal half of CA1 neurons [49]. Given that the amelioration of neuron loss upon memantine treatment seems to occur mainly in the distal CA1, this might suggest that the rescue in object recognition memory is associated with this hippocampal subfield, which is supported by the correlation analysis. As we also detected a positive correlation between neurogenesis rate and NOR performance, it is likely that other brain areas such as dentate gyrus or entorhinal cortex [50] contribute to this phenotype. In addition to beneficial effects on object recognition memory, chronic memantine treatment also improved spatial learning in the Morris water maze in Tg4-42^hom^ mice. An amelioration of spatial learning deficits in this task has been shown in several models such as APP23 [12], APP/PS1-A246E [13], or 3xTg [15, 17], in the latter model even when the 3-month treatment period was initiated in aged animals at the age of 15 months [15]. In contrast, subchronic treatment with daily intraperitoneal memantine injections of 10 mg/kg reversed memory deficits in fear-conditioning and spontaneous alternation tasks in young 5XFAD mice at 6–7 months of age, but failed to rescue memory deficits in 12–15-month-old mice with more advanced pathology [39].

While there is substantial information on the effects of memantine on learning and memory in a variety of transgenic AD mouse models, only limited data with regard to motor performance is available. Sensorimotor deficits seem to be a frequent pathological alteration in AD, although they are relatively less studied [51]. Changes in motor behavior seem to occur already quite early in the disease process [52], and deficits in motor performance have been also described in a variety of transgenic mouse models [25, 53], including Tg4-42 [54]. In good agreement with our results, oral memantine treatment did not result in improved rotarod performance in the Ts65Dn mouse model of Down syndrome [55] or the G93A SOD1 mutation model of amyotrophic lateral sclerosis [56]. On the other hand, low-dose memantine treatment of a Morbus Huntington mouse model, expressing a yeast artificial chromosome containing 128 CAG repeats, results in improvement performance in the rotarod task [57], and a small pilot trial showed improved motor scores in Huntington’s disease patients treated with 20-mg/kg memantine [58]. While we did not observe an improvement in the rotarod task, deficits in the balance beam are completely rescued. While both tasks assess balance and motor functions, the balance beam task seems to provide a more sensitive measure of certain subtle motor capabilities [59].

Memantine has been described to exert protective effects against β-amyloid-induced neurodegeneration, as vehicle-treated rats with intrahippocampal injections of Aβ_1-40_ showed a more pronounced neuronal damage in the CA1 subfield compared with rats receiving memantine [10]. In addition, memantine-treated rats injected with Aβ_1-40_ or Aβ_1-42_ showed a better preservation of cholinergic neurons in the basal forebrain compared to untreated control animals [60, 61]. While most of the classical transgenic AD mouse models with extracellular plaque deposition do not show overt hippocampal neuron loss [40, 62], Tg4-42^hom^ mice show a significantly reduced number of CA1 pyramidal neurons at 6 months of age [19]. While an in-depth analysis of the underlying molecular mechanisms is beyond the scope of the current study, the observation of a significantly ameliorated CA1 neuron loss in drug-treated Tg4-42^hom^ mice supports the assumption of a neuroprotective action of memantine.

Tg4-42^hom^ mice at 6 months of age also present with a strongly reduced neurogenesis rate in the DG [21], a phenotype that is also observed in a variety of transgenic AD mouse models based on *APP* overexpression [63]. In addition to a decreased CA1 neuron loss, a partial restoration of impaired neurogenesis was observed in 6-month-old Tg4-42^hom^ mice upon chronic memantine treatment. It has been previously shown that memantine treatment promotes neurogenesis in adult rodent brain [64–67]. This effect was observed even after a single intraperitoneal injection of a high dose (50 mg/kg) of memantine and, importantly, new-born cells differentiated into mature granule cells [65]. In other studies, a significant positive correlation between performance in spatial memory tasks and the number of young mature neurons could be established [68].

In conclusion, chronic memantine treatment of homozygous Tg4-42 mice not only improves behavioral performance in learning and memory tasks but also diminishes CA1 hippocampal neuron loss and ameliorates impaired adult dentate gyrus neurogenesis. While the use of memantine is currently only approved in moderate to late AD, our preclinical results might suggest also beneficial effects in earlier disease stages and support thorough evaluation of memantine in early or mild AD. This has not been studied yet in detail in a clinical setting, and definitive long-duration trials in mild AD are needed to establish whether starting memantine treatment earlier would be beneficial and safe in the long term [34].

## Electronic Supplementary Material

ESM 1 (PDF 818 kb)
