# Supplementary material for: Chronic Memantine Treatment Ameliorates Behavioral Deficits, Neuron Loss, and Impaired Neurogenesis in a Model of Alzheimer’s Disease
Source: Mol Neurobiol. 2020 Sep 10;58(1):204–16. doi: 10.1007/s12035-020-02120-z (PMC7695672; doi:10.1007/s12035-020-02120-z)
Supplement: Supplementary file 1 — (PDF 818 kb) [file 12035_2020_2120_MOESM1_ESM.pdf]

Supplemental Information Stazi & Wirths:

"Chronic memantine treatment ameliorates behavioral deficits, neuron loss and impaired neurogenesis in a model of Alzheimer's disease"

(Molecular Neurobiology)

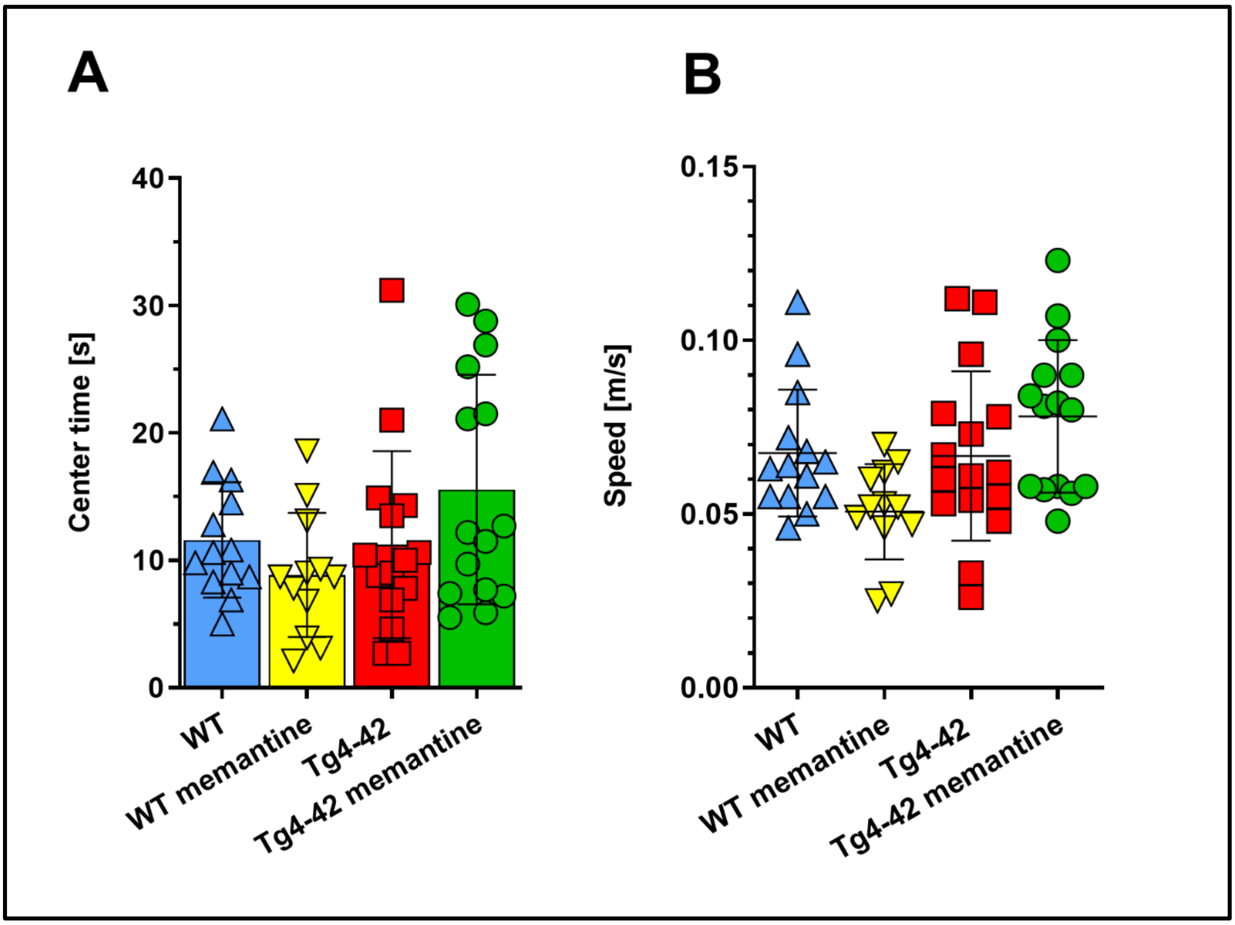

Supplemental Fig. S1: No significant differences in the time spent in the center of the open field paradigm or in the average speed were detected among the different genotypes.

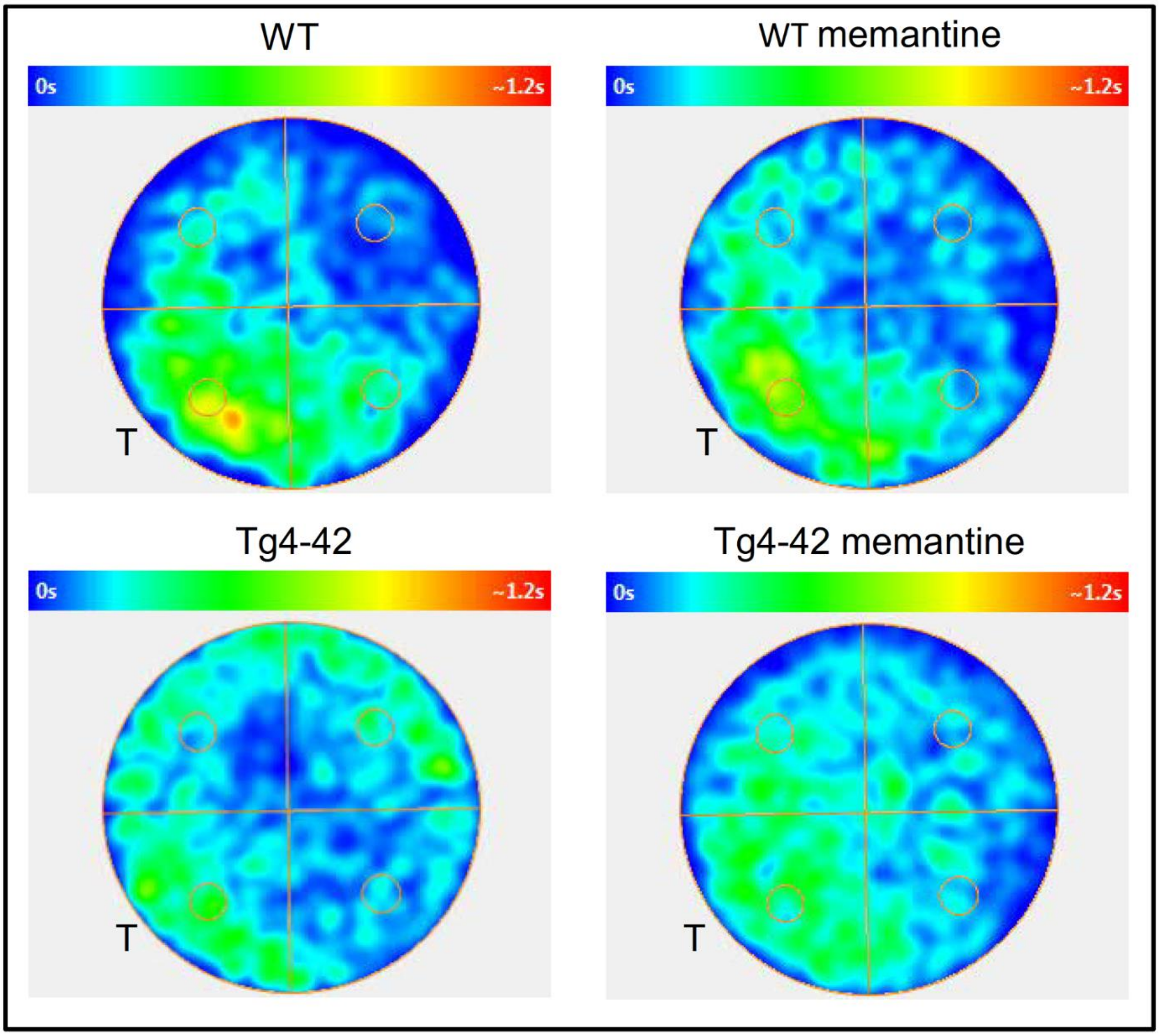

Supplemental Fig. S2: Merged occupancy plots illustrating performance of the entire groups in the probe trial in the Morris water maze paradigm. T – target quadrant

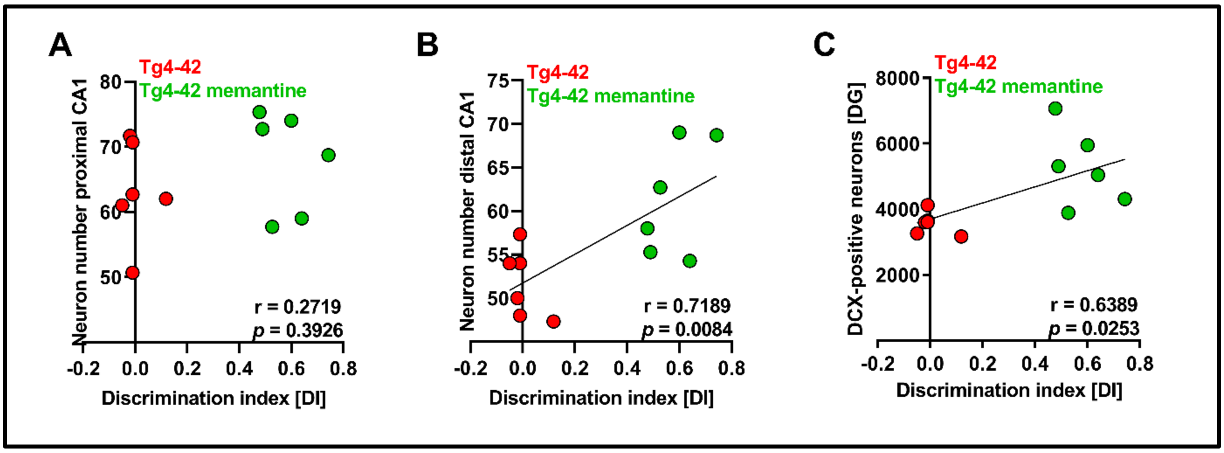

Supplemental Fig. S3: While no significant correlation could be established between the Discrimination index (DI) in the Novel Object Recognition task and CA1 neuron number in the proximal part (Pearson r = 0.2719, $p = 0.3926$) (**A**), a highly significant correlation was detected between DI and the distal CA1 neuron number (Pearson r = 0.7189, $p = 0.0084$) (**B**). The same holds true for the correlation between neurogenesis rate expressed by the number of DCX-positive neurons in the dentate gyrus (DG) and DI (Pearson r = 0.6389, $p = 0.0253$) (**C**).
